# Reply: Antibiotic prophylaxis in palliative patients with cirrhosis: Stewardship or gatekeeping?

**DOI:** 10.1097/HC9.0000000000000312

**Published:** 2023-11-22

**Authors:** Alastair O'Brien, Dominic Crocombe

**Affiliations:** 1 UCL Division of Medicine, London, UK; 2 UCL Institute for Liver & Digestive Health, Royal Free Campus, London, UK

We read with interest Haddadin et al’s letter and wholeheartedly support their innovation and leadership in palliative interventional trials in advanced liver disease. We are grateful to them for highlighting important work from this setting that we did not cite in our review. However, we disagree that our recommendations are not supported by evidence. Antimicrobial resistance (AMR) represents a global threat and the ATTIRE trial demonstrated that in the United Kingdom, clinicians may be prescribing antibiotics to double the number of hospitalized patients with advanced cirrhosis than to those who actually have evidence of infection.^1^ Crucially, this practice was not associated with any improvement in outcomes.^2^ We believe the totality of the evidence currently supports a “call to arms” to rationalize prescribing these life-saving drugs to preserve them for future use.

Furthermore, we do not view not prescribing prophylactic antibiotics to patients when there is insufficient evidence of benefit in doing so as marginalization. To be clear, in no way are we suggesting that any patient should be denied safe and effective treatments based on the etiology or severity of their disease. On the contrary, we are questioning the safety and effectiveness of prescribing prophylactic antibiotics beyond what is supported by existing evidence. Like Haddadin et al, we strongly encourage well-designed and appropriately powered clinical trials in this area to guide practice. With this in mind, we have now completed recruitment to the ASEPTIC trial^3^ and await the results in 2025.

The systematic reviews cited by Haddadin et al do highlight the increased incidence of peritonitis in patients with long-term abdominal drains (LTADs) for cirrhotic versus malignant ascites (17.4% vs 5.9%). However, this does not necessarily mean that prophylactic antibiotics will improve the outcomes, including patient-reported outcomes, for these patients. We look forward to the results from the REDUCe2 study comparing palliative LTADs with large-volume paracentesis in refractory cirrhotic ascites. The authors have pragmatically chosen to prescribe prophylactic antibiotics to all participants; thus, evaluating the safety and effectiveness of this practice will be a challenge. The consensus document quoted in support of this decision highlights that, although the risk of peritonitis is high in this patient group, “there are no evidence-based guidelines on use of prophylactic antibiotics in setting of LTADs.”^4^ We also note in the feasibility trial for REDUCe2 that the baseline rates of previous spontaneous bacterial peritonitis (SBP) for the LTAD and large-volume paracentesis groups were 7% and 13%, respectively, and the incidence of peritonitis during the 12-week trial was 6% and 11%, respectively, despite all participants taking prophylactic antibiotics.^5^ It is not clear to us whether AMR is an outcome of interest in this trial. To conclude, Haddadin et al justly warn that in the absence of evidence, “withholding treatment could have unintended consequences”; our concern is that the same is also true of giving treatments beyond the evidence. We need a higher level of evidence to endorse antibiotic prescribing beyond current evidence-based recommendations: antibiotic prescribing is not a zero-sum game.
